# Valproic Acid Inhibits the Release of Soluble CD40L Induced by Non-Nucleoside Reverse Transcriptase Inhibitors in Human Immunodeficiency Virus Infected Individuals

**DOI:** 10.1371/journal.pone.0059950

**Published:** 2013-03-28

**Authors:** Donna C. Davidson, Giovanni Schifitto, Sanjay B. Maggirwar

**Affiliations:** 1 Department of Microbiology and Immunology, University of Rochester School of Medicine and Dentistry, Rochester, New York, United States of America; 2 Department of Neurology, University of Rochester School of Medicine and Dentistry, Rochester, New York, United States of America; Centro de Biología Molecular Severo Ochoa (CSIC-UAM), Spain

## Abstract

Despite the use of highly active antiretroviral therapies (HAART), a majority of Human Immunodeficiency Virus Type 1 (HIV) infected individuals continually develop HIV – Associated Neurocognitive Disorders (HAND), indicating that host inflammatory mediators, in addition to viral proteins, may be contributing to these disorders. Consistent with this notion, we have previously shown that levels of the inflammatory mediator soluble CD40 ligand (sCD40L) are elevated in the plasma and cerebrospinal fluid (CSF) of HIV infected, cognitively impaired individuals, and that excess sCD40L can contribute to blood brain barrier (BBB) permeability *in vivo*, thereby signifying the importance of this inflammatory mediator in the pathogenesis of HAND. Here we demonstrate that the non-nucleoside reverse transcriptase inhibitor (NNRTI) efavirenz (EFV) induces the release of circulating sCD40L in both HIV infected individuals and in an *in vitro* suspension of washed human platelets, which are the main source of circulating sCD40L. Additionally, EFV was found to activate glycogen synthase kinase 3 beta (GSK3β) in platelets, and we now show that valproic acid (VPA), a known GSK3β inhibitor, was able to attenuate the release of sCD40L in HIV infected individuals receiving EFV, and in isolated human platelets. Collectively these results have important implications in determining the pro-inflammatory role that some antiretroviral regimens may have. The use of antiretrovirals remains the best strategy to prevent HIV-associated illnesses, including HAND, however these drugs have clear limitations to this end, and thus, these results underscore the need to develop adjunctive therapies for HAND that can also minimize the undesired negative effects of the antiretrovirals.

## Introduction

Human Immunodeficiency Virus Type 1 (HIV) – Associated Neurocognitive Disorders (HAND) are now found in approximately 50% of infected individuals [1] and are associated with the loss of normal neuron function leading to behavioral, motor, and cognitive deficiencies [2]–[5]. Despite the use of highly active antiretroviral therapies (HAART), which are able to efficiently control viral load, the prevalence of these disorders continues to rise as individuals live longer and more people are exposed to the current therapies [1], which largely fail to control the viral impact on the central nervous system (CNS). Although the progression of HAND seems to have shifted with use of HAART, with milder forms of impairment now more common than rapidly progressing dementia [1], there remains a lack of effective adjunct therapies to address this aspect of the disease.

Infiltration of the CNS by activated monocytic cells through a compromised blood brain barrier (BBB) is believed to be the main factor involved in neuronal dysfunction, which occurs as a result of excess inflammation in the brain that is progressively neurotoxic [6]. Consistent with this notion, we previously demonstrated that the inflammatory mediator soluble CD40 ligand (sCD40L; also known as CD154), is present at significantly higher levels in both plasma and cerebrospinal fluid (CSF) samples of HIV infected, cognitively impaired individuals [7] as compared to their infected, non-cognitively impaired counterpart. In addition, we recently reported that the HIV transactivator of transcription (Tat) alone is sufficient to stimulate the release of sCD40L *in vivo*, in a manner that promoted increased BBB permeability [8]. This effect was found to be CD40L-dependent and required the presence of platelets, the main source of sCD40L [8], [9]. Interestingly, it has been noted that decline of platelet count is correlated with an increased risk of HIV-associated dementia [10], and was found to predict brain injury in a large Multicenter AIDS Cohort study [11]. Taken together, it is plausible that aberrant platelet activation followed by increased clearance of the activated platelets is playing a large role in the etiology of these disorders. Furthermore, it was recently demonstrated that the receptor for CD40L, CD40, is upregulated during HIV infection, presumably via exposure to Tat [7], in a manner that promotes brain microvascular endothelial cell activation, monocyte recruitment [12], [13] and microglial activation [12], thus implying an important role for this receptor/ligand pair in the pathogenesis of HAND.

Interestingly, it has also been demonstrated that HAART drugs themselves have the ability to induce inflammation [14]–[16], while some commonly used antiretrovirals have been shown to possess the ability to activate platelets [17]–[19], which could therefore contribute to the progression of HAND. Taken all together, we sought to determine if common antiretrovirals could induce the release of sCD40L, and therefore potentially augment the pathogenesis of HAND. Indeed, we now show that non-nucleoside reverse transcriptase inhibitors (NNRTIs), but not other classes of antiretrovirals, can induce the release of sCD40L by directly activating platelets. We have also previously demonstrated that treatment of platelets with the clinically used mood stabilizer valproic acid (VPA) is able to attenuate the release of sCD40L, due to the antiplatelet activity of this drug [20], which has also displayed neuroprotective effects in the context of HIV infection [21], [22] and improved BBB integrity in other models [23]. Thus, we evaluated the ability of VPA to block the antiretroviral-induced release of sCD40L from platelets in an effort to further evaluate the potential of VPA as an adjunct therapy in HAND.

## Materials and Methods

### Ethics Statement

Whole blood was obtained from healthy male and female donors, and all patients gave written consent for all procedures, in accordance with the Declaration of Helinski, which were approved by the University of Rochester Research Subjects Review Board.

### Reagents and Antibodies

Antibodies against total GSK3β were purchased from Santa Cruz Biotechnology, Inc. (Santa Cruz, CA), and phospho(Ser-9)- GSK3β, p38 MAPK, phospho-p38 MAPK (Thr180/Tyr182), SAPK/JNK, and phospho- SAPK/JNK antibodies were all purchased from Cell Signaling (Danvers, MA). All antiretrovirals used for the *in vitro* studies were obtained through the NIH AIDS Research and Reference Reagent Program, Division of AIDS, NIAID, and NIH (Efavirenz, EFV, #4624; Nevirapine, NEVP, #4666; Abacavir, ABCV, #4680; Lamivudine, LAMV, #8146; Lopinavir, LOPV, #9481; and Ritonavir, RITV, #4622). Valproic acid was purchased from Sigma-Aldrich (St. Louis, MO).

### Patient Material

sCD40L levels were analyzed in the plasma of control or HIV infected individuals using ELISA. These patients (control, n = 12; efavirenz, n = 13; lopinavir, n = 10) were recruited in a previous study in which blood samples were periodically drawn before and after treatment with VPA (250 mg twice a day orally) and plasma samples were cryo-preserved [24]. The demographics, baseline clinical variables, and inclusion and exclusion criteria of the study subjects have been described [24]. Concomitant drug use was limited, as subjects receiving alternative investigational drugs within the previous 30 days or those taking medication known or suspected to interfere with drugs metabolized by the CYP isoenzyme system were excluded. The baseline clinical variables of patients include viral load <400 copies/mL, and mean CD4+ cell count 434+303.4 cells/µL. All patients were on a stable antiretroviral regimen containing efavirenz (EFV) or nevirapine (NEVP) and/or nucleoside reverse transcriptase inhibitors for at least 4 weeks before and during the entire period (7 days) of these studies, as described [24]. All patients gave written consent for all procedures, which were approved by the University of Rochester Research Subjects Review Board.

### Isolation of Human Platelets

Whole blood was obtained from healthy male and female donors, under University of Rochester IRB approval and with written informed consent in accordance with the Declaration of Helinski, by venipuncture into vacutainer tubes containing buffered sodium citrate (BD Biosciences, Franklin Lakes, NJ). Whole blood was then sequentially centrifuged to collect a purified platelet concentrate as described [25]. Platelet purity was determined to be >99%.

### ELISA

Soluble CD40L was measured in plasma samples derived from HIV infected individuals or supernatants from purified human platelets (9×10^7^ cells/sample) treated with antiretrovirals (5 µM of each drug in combinations indicated in the figures) using a human CD40L ELISA kit (R&D Systems, Minneapolis, MN) as outlined earlier [7]. The concentrations of sCD40L (pg/mL) are presented as a mean (+SEM) of four replicates for each sample. The values were then compared via paired t-test (patient samples before and after VPA treatment) or one-way ANOVA followed by Bonferroni’s test for multiple comparisons, which indicated statistical significance as *p<0.05, **p<0.01, and ***p<0.001.

### Immunoblot Assay

Isolated human platelets were treated with antiretroviral drugs, as indicated, for 1 hour at 37°C. For experiments involving VPA, platelets were treated for 5 minutes with VPA alone prior to the addition of antiretrovirals. Whole cell lysates were then prepared in ELB buffer (50 mM HEPES (pH 7), 250 mM NaCl, 0.1% Nonidet P-40, 5 mM EDTA, 10 mM NaF, 0.1 mM Na_3_VO_4_, 50 µM ZnCl_2_, supplemented with 0.1 mM PMSF, 1 mM DTT, and a mixture of protease and phosphatase inhibitors) and cellular debris was removed by high-speed centrifugation. Following separation via SDS-PAGE, protein was electrophoretically transferred to Hybond ECL nitrocellulose membrane (GE Healthcare Bio-Sciences Corporation, Piscataway, NJ, USA). The membranes were then analyzed with the indicated antibodies and bound antibodies were detected using infrared-conjugated secondary antibodies (Li-Cor Biosciences, Lincoln, NE), followed by visualization using a Li-Cor Odyssey Infrared Imaging System (Li-Cor Biosciences, Lincoln, NE). Densitometry was then performed on the resulting bands using Image J software (NIH, Bethesda, MD). The change in phosphorylation for each protein was determined as the percentage of phosphorylated molecules against total protein for each sample and fold change was subsequently calculated as compared to non-treated samples.

## Results

### Efavirenz, a Non-nucleoside Reverse Transcriptase Inhibitor (NNRTI), Results in Accumulation of sCD40L in HIV Infected Individuals

**Figure 1:**
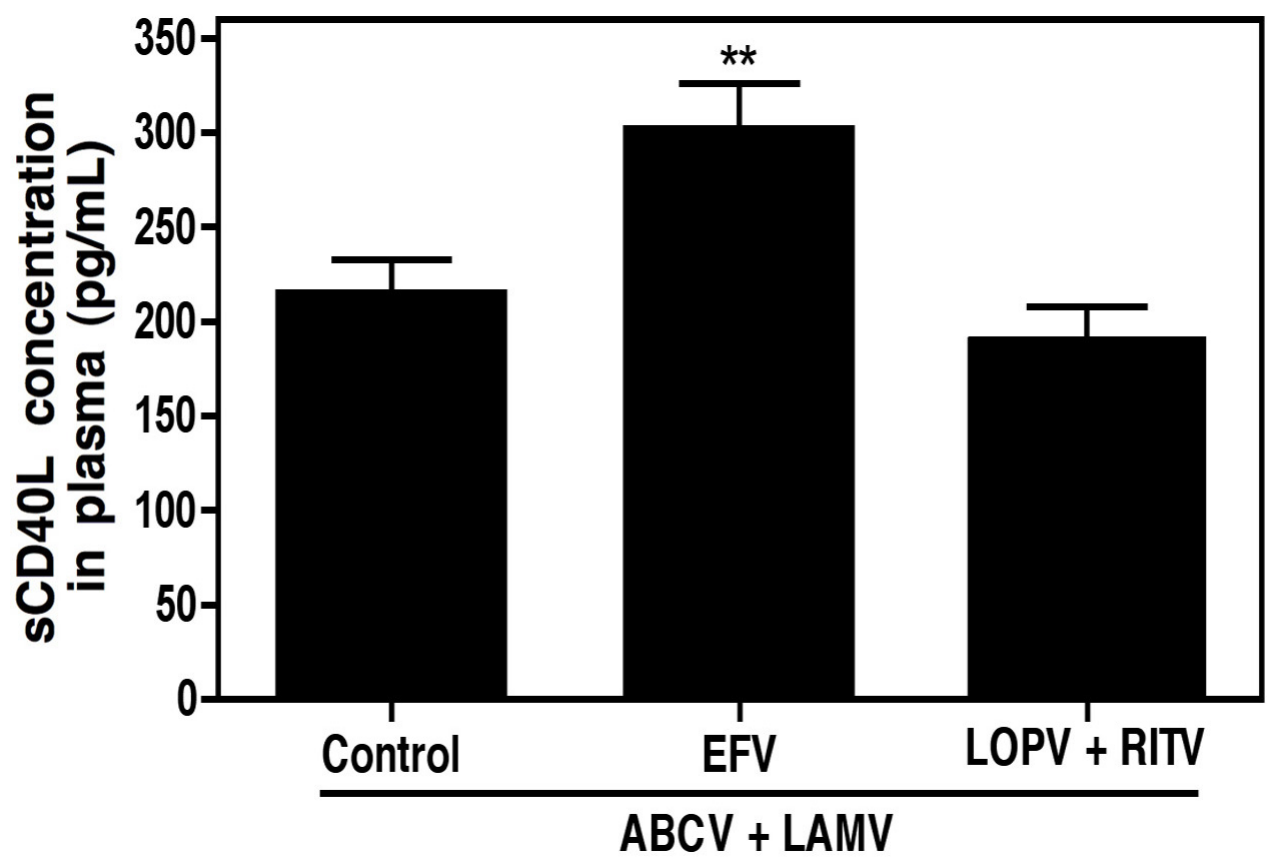
Efavirenz, a non-nucleoside reverse transcriptase inhibitor (NNRTI), results in accumulation of sCD40L in HIV infected individuals. Plasma sCD40L levels were analyzed in HIV infected individuals receiving combination antiretroviral therapy including NRTIs (abacavir, ABCV, and lamivudine, LAMV; Control, n = 12) and either an NNRTI (efavirenz, EFV; n = 13) or protease inhibitor cocktail (lopinavir, LOPV, and ritonavir, RITV; n = 10). The values were compared via one-way ANOVA followed by Bonferroni’s test for multiple comparisons, which indicated statistical significance as **p<0.01.

As discussed above, we previously observed an increase in plasma levels of sCD40L in HIV infected, cognitively impaired individuals that were receiving antiretroviral drugs, as compared to infected, non-cognitively impaired counterparts [7]. In an effort to determine whether common antiretrovirals may be responsible for excess inflammation found in HAND individuals, plasma concentrations of sCD40L were measured in HIV infected individuals receiving combination antiretroviral therapy. Patients receiving regimens consisting of the nucleoside reverse transcriptase inhibitors abacavir and lamivudine (ABCV+LAMV) alone were considered the control group, and the mean (±SEM) plasma concentration of sCD40L for this group was determined to be 216.7±15.9 pg/mL. Additionally, patient groups receiving either the NNRTI efavirenz (EFV) or the protease inhibitor (PI) lopinavir (LOPV, with a ritonavir boost, RITV) in conjunction with this ABCV+LAMV backbone were also analyzed for plasma sCD40L concentrations. Combination therapy containing an NNRTI resulted in a significant increase in plasma sCD40L concentrations, 303.8±22.0 pg/mL, whereas patients receiving a PI in addition to the NRTIs displayed plasma sCD40L concentrations similar to the control group, 189.0±18.8 pg/mL (Figure 1). These data suggest that NNRTI administration in patients induces platelet activation, either directly or indirectly.

### Non-nucleoside Reverse Transcriptase Inhibitors Induce sCD40L release directly from Isolated Human Platelets

**Figure 2:**
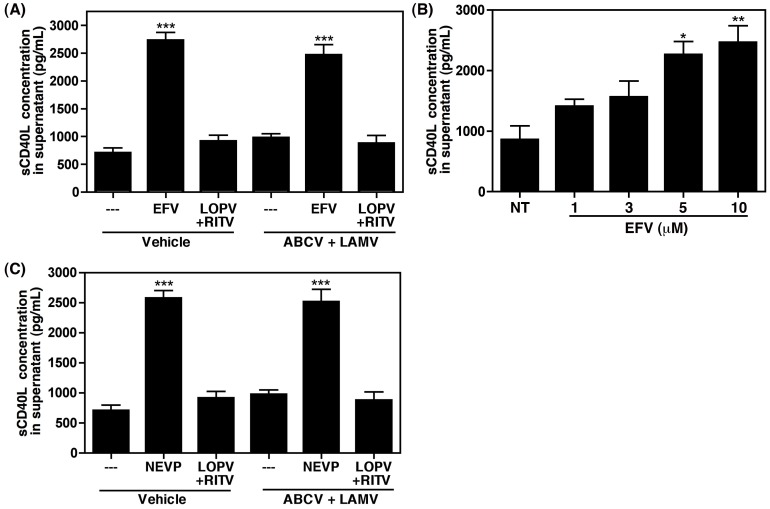
Non-nucleoside reverse transcriptase inhibitors (NNRTIs) induce sCD40L release from washed human platelets. (A) Platelets isolated from healthy donors were treated as indicated and analyzed via ELISA specific for sCD40L. Efavirenz (EFV), but not the protease inhibitors lopinavir and ritonavir (LOPV+RITV), induce sCD40L release, indicating that EFV directly activates platelets. (B) EFV induces sCD40L release in a dose dependent manner in washed human platelets, with significance achieved at physiologically relevant concentrations. (C) The NNRTI nevirapine (NEVP) also induces the release of sCD40L directly from isolated human platelets. In panels A – C values were compared via one-way ANOVA followed by Bonferroni’s test for multiple comparisons, which indicated statistical significance as *p<0.05, **p<0.01, and ***p<0.001.

To determine whether platelets are directly activated by EFV, or other antiretroviral drugs, we next treated human platelets isolated from healthy donors with the same antiretroviral combinations described above. Neither NRTIs nor PIs were able to induce the release of sCD40L directly from platelets, while EFV was able to stimulate the release of a significant amount of sCD40L (Figure 2A). The direct stimulation of platelets by EFV was found to be dose dependent, with significance reached at concentrations (5 and 10 µM) that mimic those found in patients receiving the standard dose [24], [26] (Figure 2B). In addition to EFV, the NNRTI nevirapine (NEVP) also induced the release of sCD40L from platelets (Figure 2C), indicating that commonly used NNRTIs directly activate platelets.

### Inhibition of EFV-induced Activation of GSK3β via Valproic Acid Attenuates Release of sCD40L both in vitro and in vivo

**Figure 3:**
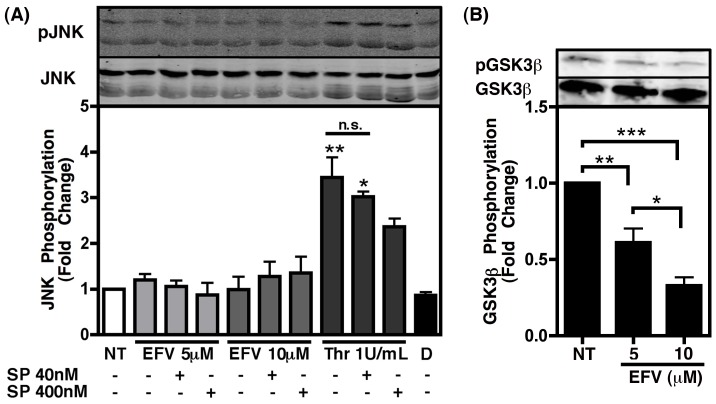
Efavirenz does not alter JNK phosphorylation status in platelets, but does induce activation of GSK3β. Washed human platelets were treated as indicated for 1 h. Graphs indicate fold change in phosphorylation as compared to non-treated (NT) and determined via densitometry of immunoblots (representative blots shown above corresponding treatments), with phospho -JNK or -GSK3β quantification determined as a percentage of the total amount of each protein for each sample. (A) EFV does not induce activation of JNK, while thrombin treatment, as a positive control, induced phosphorylation of this kinase, indicating activation. This was partially reversed when treated in the presence of increasing amounts of the JNK inhibitor SP600125 (SP). Values were compared using an one-way ANOVA followed by Bonferroni’s test for multiple comparisons, which indicated statistical significance as *p<0.05 and **p<0.01 compared to all other conditions. N.S. indicates not significant; D indicates DMSO as a vehicle control. (B) EFV treatment resulted in dephosphorylation of GSK3β at the inhibitory phosphorylation site (Ser9), indicating activation of this molecule in platelets. One-way ANOVA followed by Bonferroni’s test for multiple comparisons indicated statistical significance as *p<0.05, **p<0.01, and ***p<0.001.

In an effort to determine the mechanism by which NNRTIs are able to directly stimulate platelets, we tested the stimulation of several kinases previously reported to be involved in platelet activation, including c-Jun N-terminal kinase (JNK) [27], p38 [28], and glycogen synthase kinase 3 beta (GSK3β) [20]. Interestingly, EFV treatment of isolated platelets did not significantly increase phosphorylation of JNK, while thrombin was able to induce such an effect in a manner that was dose dependently inhibited with the JNK inhibitor SP600125, thus indicating that EFV does not stimulate JNK activity in platelets (Figure 3A). Similarly, EFV did not induce any changes in the phosphorylation status of p38 (data not shown).

**Figure 4:**
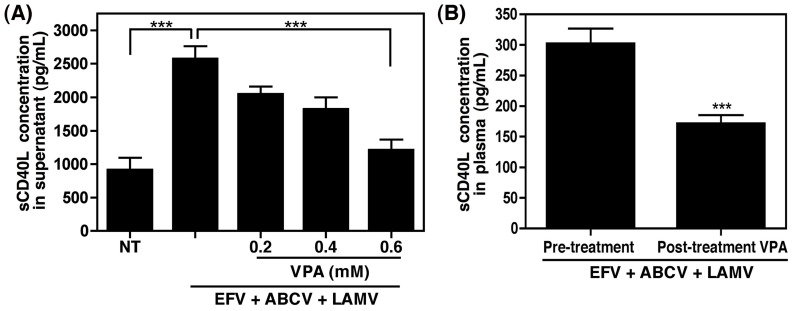
VPA inhibits EFV-induced sCD40L release from platelets both *in vitro* and in HIV infected individuals. (A) Platelets isolated from healthy donors were treated with 5 µM EFV in the presence of NRTIs abacavir and lamivudine (ABCV and LAMV, respectively), and increasing amounts of VPA. The highest dose of VPA, which is equivalent to that measured in patients receiving the standard dose, significantly inhibited the EFV-induced release of sCD40L. Values were compared via one-way ANOVA followed by Bonferroni’s test for multiple comparisons, which indicated statistical significance as ***p<0.001. (B) Plasma sCD40L levels were analyzed in HIV infected individuals receiving combination antiretroviral therapy including EFV (n = 11) prior to initiation of VPA therapy and after 7 days of VPA treatment (250 mg twice a day orally). VPA treatment significantly reduced the amount of sCD40L found in the plasma of these patients. Values were compared using a paired t-test with statistical significance indicated as ***p<0.001.

We previously demonstrated that the multifaceted kinase GSK3β was activated in platelets in response to platelet activating factor, while inhibition of this kinase with a known GSK3β inhibitor, valproic acid (VPA), attenuated the release of sCD40L via altered cytoskeletal rearrangement [20]. Thus, we tested the ability of EFV to induce activation of GSK3β in isolated platelets, and observed a dose dependent decrease in the inhibitory phosphorylation of this kinase, indicating that EFV stimulates GSK3β in platelets (Figure 3B). Interestingly, it has been shown by our group that VPA, a mood stabilizer that is used clinically, may have the potential to serve as an adjunct therapy for HAND, demonstrating a trend toward improved cognitive performance when tested in a controlled pilot patient study [22]. Furthermore, this drug has been tested in a mouse model of HIV encephalitis and demonstrated neuroprotective effects [21]. Taken together with the previously demonstrated antiplatelet activity of VPA [20], we next examined whether this drug would inhibit EFV-induced sCD40L release from platelets *in vitro*. Co-administration of EFV (5 µM) with VPA demonstrated a dose-dependent inhibition of sCD40L release, at concentrations of VPA similar to those measured in the plasma of patients receiving the standard dose [22], [24] (Figure 4A). Consistently, when administered to HIV infected patients (250 mg twice a day orally) receiving a combination antiretroviral regimen including EFV, VPA was able to significantly alleviate the EFV-induced increase in plasma sCD40L concentrations after 7 days of treatment, with sCD40L levels dropping from 301.8±25 pg/mL to 170.9±14.0 pg/mL following treatment (Figure 4B). Collectively these data highlight the therapeutic potential of this drug for HAND, as well as other inflammatory conditions in which sCD40L is implicated.

## Discussion

Neurocognitive disorders persist in approximately 50% of HIV infected individuals despite HAART [29], thus highlighting the need for the development of effective therapies to address this continual problem. Progressive deterioration of neuronal function and subsequent neurotoxicity is thought to be caused, at least in part, by an excessive inflammatory environment created within the CNS by activated cells of myeloid origin [30], [31]. Given that infection alone is enough to stimulate this type of inflammatory environment, further induction of pro-inflammatory mediators by common antiretrovirals would therefore prove to be an undesirable effect. Here we demonstrate that NNRTIs are able to stimulate the release of sCD40L, which we previously demonstrated to be upregulated in HIV infected patients with cognitive impairment as compared to those without [7]. We also previously observed that CD40L is able to synergize with HIV Tat in a manner that promotes aberrant activation of monocytes and resultant neurotoxicity [7], suggesting that any further stimulation of sCD40L release following treatment with NNRTIs could negatively contribute to the pathogenesis of HAND. This is especially important to consider when taking into account that the level of impairment is actually more strongly correlated to the number of activated macrophage and microglia within the CNS than the number of HIV infected cells or level of viral RNA [32], [33], thus highlighting the potential hazard of additional pro-inflammatory stimulation.

To this end, increased infiltration of the BBB by activated leukocytes, and subsequent induction of inflammation, is thought to be one of the largest contributing factors in the development of neurotoxicity and HAND. Consistent with this notion, we have demonstrated previously that excess sCD40L contributes to BBB permeability in the context of HAND [8]. Taken with the data presented herein, it is plausible that antiretroviral regimens that induce sCD40L are capable of contributing to the deterioration of the BBB, and hence, aid in the pathogenesis of HAND, in addition to other HIV-associated illnesses that occur as a result of chronic inflammation, platelet activation, and subsequent release of sCD40L.

The data presented here are in agreement with other previous reports that have noted an increase in sCD40L concentrations following onset of HAART therapy. For example, Wolf *et al.* found that antiretroviral therapy was able to significantly reduce markers of endothelial activation, such as soluble VCAM-1 and ICAM-1, however, markers of platelet activation, sCD40L and soluble P-selectin, remained significantly higher than healthy controls regardless of treatment, and both of these markers were increased following onset of HAART therapy that included an NNRTI [34]. Furthermore, Sipsas *et al.* noted a twofold increase in sCD40L concentrations in HIV positive individuals over healthy controls, which jumped to threefold higher following 8 to 12 months of HAART [14]. They reason that this increase in sCD40L is likely due to increased CD4+ T cell counts, since T cells express CD40L on their surface and subsequent cleavage contributes to the circulating sCD40L pool. While it is possible that the increased presence of CD4+ T cells would lead to a slight increase in the amount of T cell derived sCD40L, platelets are the main source of the sCD40L found in the plasma [8], [9], suggesting that increases in the plasma content of sCD40L would be a result of aberrant platelet activation. Taken together with the data presented here, it is probable that platelet activation as a result of NNRTI exposure is contributing to increased sCD40L concentrations in HIV infected individuals receiving an NNRTI.

It is also noteworthy that abacavir, an NRTI, has been demonstrated previously to enhance activation of platelets, thus sensitizing these cells to additional stimuli [18], [19], and was shown to modestly activate platelets directly, as measured by P-selectin surface expression [18]. Interestingly, we did not observe that platelets exposed to abacavir and lamivudine alone induce direct activation, as measured by the release of sCD40L (Figure 2). This discrepancy is likely due to the difference in the platelet activation markers analyzed, P-selectin versus sCD40L, which are differentially regulated in platelets in response to stimuli [35]. Furthermore, although abacavir seemingly does not contribute directly to the induction of sCD40L, additional stimulation of platelets by this drug resulting in upregulation of P-selectin, as indicated by Baum *et al.* [18], would further contribute to the induction of a pro-inflammatory state, as P-selectin is known to activate leukocytes and aid in the development of thrombotic complications [36], [37].

Several kinases have been implicated in the activation of platelets and the release of sCD40L, including c-Jun N-terminal kinase (JNK) [27], p38 [28], and GSK3β [20], respectively, and thus, we sought to determine if any of these kinases were activated in response to EFV. Interestingly, although JNK has been demonstrated to be involved in platelet granule secretion and thrombus formation [27], we found that JNK activation is not altered by exposure of platelets to EFV (Figure 3A). On the contrary, in agreement with published reports [38], JNK was activated following treatment with thrombin, which is also known to induce the release of sCD40L, suggesting that EFV treatment stimulates an alternative pathway to induce the release of sCD40L from the platelets. Although p38 has been implicated previously in the activation of platelets by numerous stimuli and the subsequent release of pro-inflammatory molecules (reviewed in [39]), the exact signaling pathways involved within platelets are not fully elucidated and remain controversial. It has been reported that p38 is not involved in the solubilization of CD40L in response to thrombin receptor-activating peptide [35], and similarly, we found that the phosphorylation status of this kinase was not changed in response to EFV (data not shown). Collectively these results suggest that the signaling pathways mediated by p38 during platelet activation do not contribute to the solubilization of CD40L.

Although EFV did not stimulate JNK or p38 in isolated human platelets, here we observe a significant increase in the activation of GSK3β in response to EFV treatment (Figure 3B). Interestingly, several other platelet mediators, including those known to induce sCD40L, have been shown to inhibit, rather than activate, GSK3β [40]–[42]; however, we previously found that this kinase is also activated in response to the HIV-associated inflammatory mediator platelet-activating factor (PAF) in a manner that promotes excessive sCD40L release from platelets [20]. GSK3β is a multi-faceted kinase involved in wide range of cellular activities, including cytoskeletal rearrangement, and is regulated in a very complex manner. Thus, when considered with the data presented herein, it is likely that PAF and EFV stimulate similar signaling pathways within platelets, ultimately resulting in the activation of this kinase in a manner that promotes excess sCD40L release. Indeed, we previously established that the involvement of this kinase in the process of CD40L solubilization from platelets was based on its role in cytoskeletal rearrangement during activation, and inhibition of GSK3β with VPA attenuated the release of this pro-inflammatory mediator [20].

Our group was the first to report that VPA possessed the ability to ameliorate HIV-associated neurotoxicity both *in vitro* and *in vivo* [21], [43], and demonstrated a trend toward improved cognitive performance, as well as improvements in measures of brain metabolism, when tested in a controlled pilot patient study [22]. We now show that VPA can also inhibit NNRTI-induced release of excess sCD40L in *ex vivo* treated platelets and in a small pilot population of HIV infected individuals receiving combination antiretroviral therapy including an NNRTI (Figure 4). VPA has been used clinically for years to treat bipolar disorder and epilepsy, demonstrating safety and tolerability of this drug in a clinical setting. It is also noteworthy that co-administration of VPA with efavirenz or lopinavir does not alter plasma concentrations of these drugs, indicating that the ability of VPA to decrease EFV-induced sCD40L concentrations is not due to altered or decreased plasma concentrations of this, or any other, antiretroviral [24]. Interestingly, some reports have suggested that VPA may not be well suited for use during HIV infection, as it is a histone deacetylase inhibitor and therefore may be capable of activating latent virus, and thus, increasing viral replication. However, use of VPA is not associated with an increase in viral load or HIV disease progression [44], and moreover, several studies have utilized this characteristic of VPA in patients receiving this drug in combination with high doses of HAART in an effort to deplete latent viral reservoirs, with some success [45], [46]. Due to the small sample size of the groups presented herein, we were unable to confirm these results, however, it would be interesting to evaluate immunological and virological variables such as CD4 T cell count and viral load in a larger study of patients receiving VPA as an adjunctive therapy during HIV infection.

Taken all together, addition of VPA to the therapeutic regimen of HAND patients may prove beneficial in attenuating the pathogenesis and progression of these disorders, as well as others associated with an increase in sCD40L, such as cardiovascular disease. Indeed, alternative methods to inhibit CD40L do not discriminate between sCD40L and that which is expressed on the surface of T cells, and thus have the potential to confer immunosuppression. On the contrary, antiplatelet therapies such as VPA that can dampen sCD40L release from platelets while avoiding undesirable humoral immune response inhibition, demonstrate a worthy avenue of pursuit. Interestingly, Rom *et al.* recently demonstrated that inhibition of GSK3β in monocytes inhibits their migration across an *in vitro* model of the BBB [47]. Thus, usage of GSK3β inhibitors as adjunctive therapy for HAND may prove advantageous in a multi-factorial manner, thereby warranting further investigation.

It goes without saying that HAART remains a necessary component of the AIDS epidemic that cannot be removed due to every adverse side effect, including excess pro-inflammatory stimulation. However, identifying off target effects of these drugs, as those described herein, will allow proper monitoring and determination of therapeutic regimens that can be varied accordingly based on individual response and need. Furthermore, development of novel adjunctive therapeutics to offset these effects, such as VPA, is imperative, as this remains a critical barrier in the management of HAND.
